# Pulmonary function in patients with transfusion-dependent thalassemia and its associations with iron overload

**DOI:** 10.1038/s41598-023-30784-9

**Published:** 2023-03-04

**Authors:** Kate C. Chan, Chun T. Au, Alex W. K. Leung, Albert M. Li, Chi-kong Li, Matthew M. T. Wong, Carol S. T. Li, Hang L. Cheung, Philip Fan, Siu C. Ling, Rever C. H. Li, S. Y. Ha

**Affiliations:** 1 Department of Paediatrics, Faculty of Medicine, The Chinese University of Hong Kong, 6/F, Lui Che Woo Clinical Sciences Building, Prince of Wales Hospital, Shatin, N.T., Hong Kong SAR China; 2 Laboratory for Paediatric Respiratory Research, Faculty of Medicine, Li Ka Shing Institute of Health Sciences, The Chinese University of Hong Kong, Shatin, Hong Kong SAR China; 3 Hong Kong Hub of Paediatric Excellence, The Chinese University of Hong Kong, Shatin, Hong Kong SAR China; 4 Department of Paediatrics and Adolescent Medicine, Princess Margaret Hospital, Kowloon, Hong Kong SAR China; 5 Department of Paediatrics and Adolescent Medicine, Tuen Mun Hospital, Tuen Mun, Hong Kong SAR China; 6 Department of Paediatrics and Adolescent Medicine, Queen Mary Hospital, Pok Fu Lam, Hong Kong SAR China

**Keywords:** Haematological diseases, Respiratory tract diseases

## Abstract

In patients with transfusion-dependent thalassemia (TDT), pulmonary function impairment has been reported but data are conflicting. Moreover, it remains unclear whether pulmonary dysfunction is associated with iron overload. This study aimed to evaluate the pulmonary function in patients with TDT and to investigate the associations between pulmonary dysfunction and iron overload. It was a retrospective observational study. 101 patients with TDT were recruited for lung function tests. The most recent ferritin levels (pmol/L) and the magnetic resonance imaging (MRI) measurements of the myocardial and liver iron status, as measured by heart and liver T2* relaxation time (millisecond, ms) respectively, were retrieved from the computerized medical records. Only data within 12 months from the lung function measurement were included in the analysis. The serum ferritin, and the cardiac and liver T2* relaxation time were the surrogate indexes of body iron content. The threshold of abnormality in lung function was defined as under 80% of the predicted value. 101 subjects were recruited with a mean age of 25.1 years (standard deviation (SD) 7.9 years). Thirty-eight (38%) and five (5%) demonstrated restrictive and obstructive lung function deficits, respectively. A weak correlation of FVC %Predicted and TLC %Predicted with MRI myocardial T2* relaxation time (rho = 0.32, *p* = 0.03 and rho = 0.33, *p* = 0.03 respectively) was observed. By logistic regression, MRI cardiac T2* relaxation time was negatively associated with restrictive lung function deficit (B − 0.06; SE 0.03; Odds ratio 0.94; 95% confidence interval (CI) 0.89–0.99; *p* = 0.023) after adjusting for age, sex and body mass index. Restrictive pulmonary function deficit was commonly observed in patients with TDT, and the severity potentially correlates with myocardial iron content. Monitoring of lung function in this group of patients, particularly for those with iron overload, is important.

## Introduction

Transfusion-dependent thalassemia (TDT) is a genetic disorder characterised by abnormal hemoglobin synthesis, which results in ineffective erythropoiesis, hemolysis, and severe transfusion-dependent anemia^1^. While regular blood transfusion is the mainstay of management for patients with TDT, iron overload from repeated transfusions remains a major cause of multi-organ morbidities despite the availability of iron chelation therapy^1–4^. The heart, liver, and endocrine glands are the organs known to be most frequently affected^5–7^. However, the effects on the lungs in patients with TDT are much less studied and the literature data are contradictory^8^.

Up to 80% of patients with TDT were reported to have pulmonary dysfunction^9,10^. The pulmonary abnormalities identified in previous studies include restrictive lung function deficit (13.8–76.9%)^8,11–20^, obstructive pattern (3.2–32%)^9,16–18^, and decreased diffusion capacity for carbon dioxide (2.7–85.7%)^8,14,15,17,18,20,21^. Although the pathophysiology of pulmonary dysfunction is poorly defined, iron accumulation from repeated blood transfusions has been proposed as the likely cause^8,9,22–24^. This is supported by autopsy data and post-mortem examinations, which found pulmonary iron deposition in patients with TDT who had previously received multiple transfusions^25,26^.

Serum ferritin levels provide a measure of the body’s iron status and high levels correlate with iron overload in patients with TDT^1^. Magnetic resonance imaging (MRI) is commonly used to quantify and monitor iron deposition in organs such as the heart and the liver^27^. Cardiac MRI, a widely adopted non-invasive tool for monitoring iron overload in the heart, can detect myocardial iron overload earlier than the ventricular dysfunction identified by an echocardiogram^27–30^. MR T2-star (T2*) technique can reproducibly quantify myocardial iron deposition, a lower T2* relaxation time represents higher iron overload status. Nonetheless, the relationship between this MRI marker of iron status and pulmonary dysfunction in patients with TDT remains to be ascertained. A previous study did not find a correlation between lung function deficits with cardiac or liver iron status measured by the MRI^8^. However, among patients with high serum ferritin > 2500 μg/l, a lower cardiac T2* (14.28 ± 9.99 ms vs. 31.59 ± 7.43 ms) was observed in patients with restrictive lung function abnormalities when compared to those without restrictive lung function pattern^8^. Data on the secular trend of lung function in patients with TDT is also scarce. A previous study that examined 18 adult patients with TDT, at baseline and 7 years later could not demonstrate significant differences in lung function parameters between the two time points^13^. From a longitudinal study of the pulmonary function of 30 patients with TDT, similarly none of the lung function parameters significantly changed when compared with the previous evaluation performed 10 years ago^8^. However, the subjects involved were already in their adulthood at baseline and therefore the transition from childhood to adulthood could not be evaluated^8,13^. Our group previously reported the lung function results of a group of patients with TDT at a mean age of 14.2 years^17^. Repeat assessment of this cohort would allow informative observation on the natural progression of their lung function.

In this study, we aimed (1) to identify the patterns of pulmonary function deficits in a cohort of patients with TDT; (2) to examine the correlations between pulmonary function abnormalities with serum ferritin and MRI measurements of iron content in the myocardium and the liver; (3) to compare pulmonary function test parameters between two time points in a subgroup of patients.

## Results

### Sample characteristics

One hundred and one patients were recruited (mean age: 25.1 ± SD 7.9 years) and 52 (51%) were males. The median age at the diagnosis of TDT was 0.8 years (Interquartile range (IQR): 0.5–2.0 years) and the mean duration of regular blood transfusions was 23.3 ± 8.0 years. The characteristics of the participants are shown in Table 1. No patients had clinical symptoms or signs of lung disease.

**Table 1:** Clinical characteristics of the participants (N = 101).

Characteristics	Value
Age (years)	25.1 ± 7.9
Male sex, n (%)	52 (51.5%)
Weight (kg), median (IQR)	48.1 (41.8–52.9)
Height (cm), median (IQR)	155.5 (149.0–161.9)
BMI (kg/m^2^)	20.0 ± 2.5
Age at diagnosis (years), median (IQR)	0.8 (0.5–2.0)
Age of starting regular blood transfusion (years), median (IQR)	1.0 (0.5–3.0)
Duration of blood transfusion (years)	23.3 ± 8.0
Time interval between ferritin and lung function test (days), median (IQR)	47.0 (21.0–72.0)
Serum ferritin (pmol/L), median (IQR)	4118.0 (2505.5–6459.5)
Time interval between MRI cardiac T2* and lung function test (days), median (IQR)	161.0 (107.0–243.0)
MRI cardiac T2* relaxation time (ms) (N = 47)	34.7 ± 17.9
Number of patients who had cardiac T2* < = 20 ms, n (%)	9 (19.1%)
Time interval between MRI liver T2* and lung function test (days), median (IQR)	173.5 (127.3–240.8)
MRI liver T2* relaxation time (ms), median (IQR) (N = 36)	4.7 (2.3–10.9)
Number of patients who had liver T2* < = 6.3 ms, n (%)	21 (58.3%)

Values are presented as mean ± standard deviation unless otherwise specified.

BMI, body mass index; IQR, interquartile range; MRI, magnetic resonance imaging.

### Lung function patterns of the subjects

**Figure 1:**
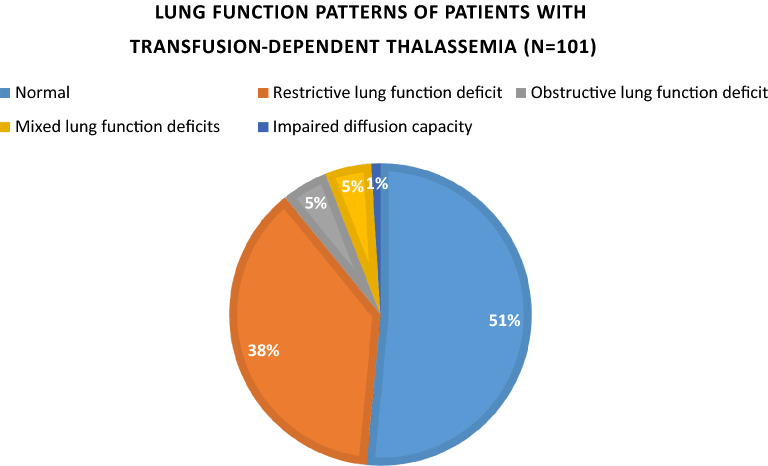
Lung function patterns of patients with transfusion-dependent thalassemia.

The distribution of lung function patterns among patients with TDT is shown in Fig. 1. Fifty-two subjects (51%) had a normal lung function. Thirty-eight (38%) and five (5%) subjects had restrictive and obstructive lung function deficits, respectively. One (1%) had impaired diffusion capacity. Five subjects (5%) had mixed lung function deficits: two had mixed restrictive and obstructive patterns, one had mixed restrictive deficit and impaired diffusion capacity, one had mixed obstructive pattern and impaired diffusion capacity, and one had mixed restrictive obstructive patterns and impaired diffusion capacity. As restrictive lung function deficit was the predominant deficit observed, the margin of error of the prevalence of restrictive lung function in our study population with TDT was estimated. The margin of error was 9.47% for a confidence interval of 95% with our current sample size.

A comparison between patients with normal lung function and those with restrictive lung function pattern is shown in Table 2. As expected, patients with restrictive lung function deficit had significantly lower FVC % predicted (75.5%, IQR: 68.0–82.0% vs. 91.0%, IQR: 87.0–94.8%, *p* < 0.001) and TLC % predicted (74.5%, IQR 67.3–78.8% vs. 91.0%, IQR 85.0–100.0%, *p* < 0.001) than those with normal lung function. As FEV_1_ and FVC are closely related, patients with restrictive lung function deficit also had significantly lower FEV_1_% predicted (77.0%, IQR: 70.0–83.3% vs. 92.0%, IQR: 84.0–98.0%, *p* < 0.001) than those with normal lung function. There were no significant differences in other characteristics such as age, gender, weight, height, BMI, duration of regular blood transfusion, and serum ferritin concentrations.

**Table 2:** Comparisons of clinical characteristics between participants with normal lung function and those with restrictive lung disease.

Characteristics	Normal (n = 52)	Restrictive (n = 38)	*P*
Age (years), mean ± SD	24.9 ± 7.8	25.6 ± 8.1	0.71
Sex, n (%)	F: 24 (46.2%) M: 28 (53.8%)	F: 20 (52.6%) M: 18 (47.4%)	0.67
Weight (kg)	45.6 (41.4–50.8)	49.3 (42.9–53.5)	0.14
Height (cm)	153.4 (148.3–159.8)	156.5 (150.0–161.9)	0.26
BMI (kg/m^2^), mean ± SD	19.7 ± 2.4	20.4 ± 2.5	0.15
Age at diagnosis (years)	0.8 (0.5–1.5)	0.7 (0.3–2.5)	0.98
Age of starting blood transfusion (years)	1.0 (0.5–3.0)	1.0 (0.5–3.0)	0.63
Duration of blood transfusion (years), mean ± SD	23.4 ± 7.8	23.5 ± 8.3	0.94
Serum ferritin (pmol/L)	3625.0 (2381.5–5983.0)	4474.0 (2349.8–6054.8)	0.55
FVC (% predicted), mean ± SD	91.9 ± 7.9	74.2 ± 9.3	< 0.001
FEV1 (% predicted), mean ± SD	92.0 ± 9.0	76.6 ± 9.1	< 0.001
FEV1/FVC (%)	88.0 (85.0–91.8)	91.0 (89.0–93.3)	0.01
FEV1/FVC (% predicted)	100.5 (96.0–104.0)	103.0 (101.0–108.0)	0.001
TLC (% predicted)	91.0 (85.0–100.0)	74.5 (67.3–78.8)	< 0.001
DLCO/VA (% predicted)	112.0 (101.0–130.5)	129.0 (113.3–141.3)	0.01
MRI cardiac T2* relaxation time (ms), mean ± SD	40.1 ± 18.2 (N = 29)	26.5 ± 13.2 (N = 14)	0.02
Number of patients having abnormal cardiac T2* < = 20 ms, n (%)	4 (13.8%)	3 (21.4%)	0.67
MRI liver T2* relaxation time (ms), median (IQR)	5.1 (2.2–10.9) (N = 24)	3.8 (2.4–12.0) (N = 9)	0.77
Number of patients having abnormal liver T2* < = 6.3 ms, n (%)	13 (54.2%)	6 (66.7%)	0.70

Values are presented as medial and interquartile range (IQR) unless otherwise specified.

*P* values by t-test, Mann–Whitney U and chi-square test for parametric, non-parametric, and categorical variables respectively.

BMI, body mass index; SD, standard deviation; FVC, forced vital capacity; FEV1, forced expiratory volume in 1 s; TLC, total lung capacity; DLCO, diffusing capacity of the lung for carbon monoxide; VA, alveolar volume; MRI, magnetic resonance imaging.

Respectively, 14 and 29 subjects with restrictive and normal lung function patterns had cardiac MRI data available within 12 months from the lung function assessment. Former group had significantly lower MRI cardiac T2* than those with normal lung function (26.5 ± 13.2 ms vs. 40.1 ± 18.2 ms, *p* = 0.02) (Table 2).

When only taking those with available MRI measurements into consideration, those with restrictive lung function deficit had a male gender predominance (64%) and significantly higher body weight and height when compared to those with normal lung function (Supplementary Table 1). There were no significant differences in other characteristics such as age, gender, BMI, duration of regular blood transfusion, and serum ferritin concentrations between those with normal lung function and those with restrictive lung function deficit.

### Correlations between lung function and surrogate indexes of body iron content

A weak correlation between MRI myocardial T2* and FVC %Predicted (rho = 0.32, *p* = 0.03) was observed (Table 3). Similarly, there was a weak correlation between MRI myocardial T2* and TLC %Predicted (rho = 0.33, *p* = 0.03). (Table 3) By logistic regression analysis, MRI cardiac T2* relaxation time was negatively and weakly associated with restrictive lung function pattern (B − 0.06, SE 0.03; Odds ratio (OR) 0.94 (95% confidence interval (CI): 0.89–0.99; *p* = 0.02) after adjusting for age, sex and body mass index (Supplementary table 3). There were 9 patients with abnormal cardiac T2* of <  = 20 ms. By logistic regression, abnormal cardiac T2*, when compared to normal, was not associated with restrictive lung function pattern (Supplementary table 4). Similarly, abnormal liver T2* or serum ferritin concentration was not associated with restrictive lung function pattern (Supplementary table 5 and 6).

**Table 3:** Correlations between lung function and surrogate indexes of body iron content.

Measurements	FVC (% Pred)	FEV1 (% Pred)	FEV1/FVC (%)	TLC (% Pred)	DLCO/VA (% Pred)
Serum ferritin	rho = − 0.04 *p* = 0.69	rho = − 0.06 *p* = 0.56	rho = − 0.06 *p* = 0.53	rho = − 0.08 *p* = 0.47	rho = − 0.19 *p* = 0.06
MRI cardiac T2* relaxation time	rho = 0.32 *p* = 0.03	rho = 0.30 *p* = 0.04	rho = 0.03 *p* = 0.86	rho = 0.33 *p* = 0.03	rho = − 0.07 *p* = 0.67
MRI liver T2* relaxation time	rho = 0.08 *p* = 0.64	rho = 0.03 *p* = 0.88	rho = -0.09 *p* = 0.60	rho = 0.09 *p* = 0.62	rho = 0.02 *p* = 0.89

Correlations were performed by Spearman’s rank correlation.

FVC, forced vital capacity; FEV1, forced expiratory volume in 1 s; TLC, total lung capacity; DLCO, diffusing capacity of the lung for carbon monoxide; VA, alveolar volume; MRI, magnetic resonance imaging.

### Longitudinal evaluation of a subgroup

This study included 23 subjects (13 males) who underwent lung function assessment 13 years ago (Supplementary Fig. 1). There were no statistically significant differences in the clinical characteristics between subjects who had longitudinal lung function data and those who did not (Supplementary Table 2). Longitudinal changes in lung function over time are shown in Table 4. DL_CO_/VA was not available at baseline and therefore only comparison in DL_CO_ was performed. The decrease in the FVC %Predicted just reached borderline statistical significance. Overall, these 23 subjects did not have significant changes in their pulmonary function over time. At baseline, four subjects had restrictive lung function deficit, three of them continued to have restrictive pattern at follow-up. Three patients who had normal lung function at baseline developed restrictive abnormality at follow-up. Further statistical analysis was not performed given the small subject number.

**Table 4:** Longitudinal changes in lung function over time (N = 23).

Characteristics	Baseline	Follow-up	*P*
Age	13.73 ± 3.80	26.79 ± 3.62	-
FVC (% predicted)	90.83 ± 12.22	86.13 ± 11.85	0.05
FEV1 (% predicted)	86.57 ± 11.59	85.48 ± 11.52	0.57
FEV1/FVC (%)	86.91 ± 4.99	87.70 ± 3.43	0.36
TLC (% predicted)	96.10 ± 16.82	88.50 ± 19.11	0.09
Log-transformed TLC	4.55 ± 0.19	4.46 ± 0.20	0.06
DL_CO_ (% predicted)	90.86 ± 22.73	93.27 ± 30.03	0.66
Log-transformed DL_CO_	4.48 ± 0.24	4.49 ± 0.07	0.93

Values are presented as mean ± standard deviation unless otherwise specified.

*P* values by paired sample *t*-test.

FVC, forced vital capacity; FEV1, forced expiratory volume in 1 s; TLC, total lung capacity; DL_CO_, diffusing capacity of the lung for carbon monoxide.

## Discussion

Our study demonstrated that restrictive lung function deficit was the most prevalent pulmonary function abnormalities seen in individuals with TDT. There was a weak correlation between myocardial T2* relaxation time on MRI measurement and FVC %Predicted as well as TLC %Predicted. The findings suggested that restrictive lung function deficit in patients with TDT may be associated with their myocardial iron content.

Our study shared a similar prevalence of restrictive lung function deficits with some published studies^8,13^. Guidotti et al. summarised the literature data on pulmonary function in patients with TDT^8^. The study results have been heterogeneous in terms of the pattern of pulmonary abnormalities observed. However, restrictive lung function pattern has been commonly reported in many of the studies with a prevalence ranging from 13.8 to 79%^8^. Although the exact pathophysiological mechanism is not known, iron accumulation in the lungs and chronic hypoxia related to chronic anemia are believed to be important factors contributing to the development of pulmonary dysfunction in patients with TDT. Patients with TDT develop systemic iron overload because of multiple blood transfusions and the absence of a regulated way to excrete iron. Iron accumulation was detected in alveolar macrophages from these patients supporting the hypothesis that increased systemic iron levels elevate pulmonary iron content^31^. This is supported by autopsy studies showing that iron was concentrated in bronchiolar epithelial and mucous glands. In patients with TDT, iron-laden macrophages and excessive sideroblasts were also noted in alveolar spaces and present in bronchoalveolar lavage, often in quantities like those observed in idiopathic pulmonary hemosiderosis, as well as lymphocytic infiltrates suggestive of alveolitis^5,32,33^. Iron accumulation could cause lung parenchymal fibrosis through the production of potentially toxic hydroxyl radicals, and hence lead to restrictive lung function deficits^34,35^. Iron deposition in the lung also causes arterial wall stiffening, which increases pulmonary vascular resistance. It exacerbates oxidative stress, which along with impairment of the arginine-NO pathway, promotes endothelial injury and contributes to the development of pulmonary arterial hypertension in transfusion-dependent thalassemia^35^. Our study is one of the few that found an association between restrictive lung function deficit and markers of iron overload. Interestingly, such correlation was observed in myocardial T2* only but not other markers such as serum ferritin or liver T2*. Another study reported a lower cardiac T2* in patients with restrictive lung function deficit when compared to those without restrictive pattern, yet the difference was observed only in patients with high serum ferritin^8^. Parakh et al. evaluated pulmonary function in 31 patients with TDT, and in those with abnormal results computed tomography (CT) of the chest and bronchoalveolar lavage (BAL) were also performed^18^. In the 15 patients with pulmonary deficit, bronchial dilatation and air trapping signifying small-airway disease were the commonest findings on chest CT, and iron-laden macrophages were demonstrated in 14 out of the 15 BAL samples^18^. Moreover, a previous study reported improvements in lung function when good control of iron status was achieved with optimal chelation therapy^13,36^. Overall, the findings supported the hypothesis that iron overload is an important culprit of pulmonary dysfunction in patients with TDT. However, the associations between pulmonary dysfunction and iron accumulation were not demonstrated in all studies^37^. In fact, some autopsy studies with small sample size did not detect any fibrosis or increase of hemosiderin in the lungs from patients with TDT^38,39^. Moreover, it is important to note that in our study although the value of cardiac T2* in the group with restrictive lung function pattern was significantly lower than that in those with normal lung function, both values were within the normal T2* category regarding the cardiac hemosiderosis. Although our correlation analysis showed a potential association between restrictive lung function and myocardial iron content, a significant association between restrictive lung function deficit and abnormal cardiac T2* was not observed in regression analysis. Therefore, more studies with larger sample size would be needed to investigate the relationship between iron overload and pulmonary dysfunction, and to elucidate the pathogenesis of lung function impairment in this group of patients.

Reduced lung function is an independent factor associated with overall and cardiovascular mortality, the observed pulmonary abnormalities are clinically relevant taking into account the enhanced life expectancy of patients with TDT^40–42^. Therefore, regular pulmonary function assessment should be incorporated in the management plan of patients with β-thalassemia major. Early detection of lung function abnormalities, timely pulmonary rehabilitation and avoidance of risk factors such as smoking and exposure to environmental tobacco smoke are important measures to slow down lung function decline. The subgroup in our study demonstrated that three out of 19 patients who had normal lung function at baseline developed restrictive lung function deficit at follow-up. However, the sample number was too small to identify risk factors of lung function decline. Further studies would be needed to study the predictive factors, long-term outcomes, and implications of the demonstrated pulmonary function abnormalities.

There were a few limitations in our study. Our study was not able to address the pathogenesis of pulmonary dysfunction in patients with TDT. Although iron overload has been the principal hypothesis, other factors were not evaluated in our study, such as the effect of chronic hypoxia, skeletal changes and abnormal chest conformation on lung growth and ventilation mechanics^8^. Moreover, our study was not able to eliminate the potential confounding effect from iron chelation therapy, while desferrioxamine has been suggested as a potential cause of lung function impairment^43,44^. Patients with iron overload were all on iron chelation therapy and very likely those with more severe iron overload were on a higher dosage of iron chelation. The use of iron chelation therapy could be a potential source of bias. However, as the data on the dosage and the length of treatment were not complete in our data collection, the therapy and compliance were also highly heterogeneous, robust evaluation of the effect of iron chelation therapy on the outcome measurement was not feasible in our study. More studies are needed to evaluate how iron chelation therapy impacts lung function. The sample size and the study power were limited by the number of patients under the care of the study centres. Only some of the patients had available MRI data for analysis which might cause potential bias. The MRI images were reported by different radiologists from different centres, which might also result into potential disagreements. The number of patients who had abnormal cardiac T2* was small and significantly limited the power of the related regression analysis. Finally, the sample size of the subgroup who had lung function reassessment was too small to evaluate the natural history of pulmonary disease and to identify risk factors of lung function decline.

## Conclusion

Restrictive lung function deficit is prevalent in patients with TDT. A weak but significant association between restrictive lung function deficit and myocardial iron accumulation was observed. As lung function decline is associated with significant morbidity and mortality, it would be important to incorporate regular pulmonary function assessment in the management plan of patients with TDT. Future studies should further evaluate the pathogenic mechanism and the long-term outcomes of pulmonary dysfunction in this particular patient group.

## Study design and methods

### Study design and participants

It was a retrospective observational study. The study period was between Jan 2013 and Jan 2015. Patients diagnosed with TDT and being followed up at the four participating hospitals (Prince of Wales Hospital, Princess Margaret Hospital, Tuen Mun Hospital, and Queen Mary Hospital) were identified. TDT was defined as the dependence on regular blood transfusions every 6 weeks or more frequent, to maintain a pre-transfusion hemoglobin level at or above 9 g/dl. Inclusion criteria were (1) age above 6 years at the time of recruitment, (2) diagnosis of TDT as defined, and (3) free from symptoms of respiratory tract illness for at least 2 weeks before lung function assessment. Exclusion criteria were any cardio-pulmonary conditions for example pulmonary hypertension, congestive heart failure, or chronic lung disease that restricted the subject from physical exertion. Written informed consent was obtained from the eligible subjects or their parents if they were younger than 18 years of age. Among the recruited patients, those who underwent lung function assessment in the same lung function laboratory 13 years ago were identified to allow the comparisons between the baseline and follow-up lung function assessment. (Supplementary Fig. 1).

### Research ethics

Ethics approval was obtained from the Joint Chinese University of Hong Kong – New Territories East Cluster Clinical Research Ethics Committee (Reference number: 2012.128). All methods were performed in accordance with the relevant guidelines and regulations.

### Data collection and anthropometric measurements

Past medical history and relevant clinical information with regards to transfusion history were obtained from the case notes. Serum ferritin, MRI cardiac T2* and liver T2* were used as surrogate indexes of body iron content^29^. The most recent ferritin levels were retrieved from the medical record. The cardiac T2* and liver T2* results were retrieved from the computerized clinical management system. Only data within 12 months from the lung function measurement were included in the analysis. The normal reference categories for cardiac T2* were: normal > 20 ms; mild 14–20 ms; moderate 10–14 ms; and severe < 10 ms. The normal reference categories for liver T2* were: normal > 6.3 ms; mild 2.7–6.3 ms; moderate 1.4–2.7 ms; and severe < 1.4 ms. The radiologists involved in the MRI reporting were blinded to the patients’ lung function measurements. The height and weight of each subject were measured for determining the body mass index (BMI). Standing height without shoes was measured using a Harpenden stadiometer (Holtain, UK) to the nearest 0.1 cm. Body weight was measured with the lightest clothing to the nearest 0.1 kg by an electronic weighing scale (Tanita BF-522, Japan).

### Lung function measurements

All subjects were invited to have lung function measurements before the scheduled transfusion. The lung function test was performed at the Prince of Wales Hospital according to the recommended standards by a trained research personnel who was blinded to the clinical status of the patients^45,46^. Spirometry was measured by a SensorMedics 2130 spirometer using the Enhanced Spirometry Program. The best of at least three technically acceptable values for forced expiratory volume in one second (FEV_1_), forced vital capacity (FVC) and flow-volume curves were selected^45,46^. Total lung capacity (TLC) was measured by body plethysmography (SensorMedics 6200) and expressed in liters corrected for body temperature, atmospheric pressure, and saturation with water vapour^45,46^. The diffusion capacity of carbon monoxide was measured by single breath technique and the values obtained were corrected for hemoglobin concentration (SensorMedics 6200 Autobox DL, Single Breath Diffusion Capacity DLco SB Program)^45,46^. The pulmonary function results were expressed as percentages of predicted normal values^47^. For this study, the threshold of abnormality was defined as under 80% of the predicted value. A restrictive deficit was defined as a reduction in TLC or FVC to less than 80%; diffusion impairment as a reduction in diffusing capacity divided by the alveolar volume (DL_CO_/VA) to less than 80%, and obstructive airway disease as reduced FEV_1_ and FEV_1_/FVC ratio to less than 80%.

### Measurement of serum ferritin concentration

Serum ferritin concentration was measured by electrochemiluminescence immunoassay technology (Cobas e 801 immunoassay analyser, Roche Diagnostics Corp, Indianapolis, IN, USA). Serum sample was mixed with a biotinylated monoclonal ferritin-specific antibody and a monoclonal ferritin-specific antibody labelled with ruthenium complex to form a sandwich complex. After the addition of streptavidin-coated microparticles, the complex became bound to the solid phase via interaction of biotin and streptavidin. The reaction mixture was aspirated into the measuring cell where the microparticles were magnetically captured onto the surface of the electrode. Unbound substances were then removed by a washing step. Application of a voltage to the electrode then induced chemiluminescent emission which was measured by a photomultiplier.

### Statistical analysis

Patient characteristics were described using frequencies for categorical variables, means and standard deviations (SD) for normally distributed continuous variables, and median and interquartile range (IQR) for skewed data. The primary outcome measure was the lung function assessment. Serum ferritin, MRI cardiac T2* and liver T2* were used as surrogate indexes of body iron content. Pulmonary function test variables were expressed as a percent of predicted value according to local references^47^. *T*-test, Mann–Whitney U, and chi-square tests (or Fisher’s exact if appropriate) were used for normally distributed, skewed, and categorical data, respectively, to assess differences in clinical characteristics between patient groups. Correlations between lung function results, serum ferritin level, and cardiac T2* and liver T2* were assessed by Spearman’s rank correlations. Logistic regression was used to assess risk factors associated with pulmonary function deficits. For the subgroup with baseline and follow-up lung function assessment performed, a paired-samples *t*-test was used to assess changes in lung function parameters over time. Statistical analyses were performed using SPSS statistical software package V.25.0 for Windows. *P* values < 0.05 were considered significant.

### Ethics approval

Some results of this study were previously presented at the European Respiratory Society International Congress 2015 and published in the congress proceedings in the European Respiratory Journal.

## Supplementary Information

Supplementary Tables.

Supplementary Figures.

## Data Availability

The datasets generated during and/or analysed during the current study are available from the corresponding author on reasonable request.

## Abbreviations

BMI: Body mass index
CI: Confidence interval
DL_CO_: Diffusing capacity of the lung for carbon monoxide
DLco/VA: Diffusing capacity of the lung for carbon monoxide divided by alveolar volume
FEV1: Forced expiratory volume in 1 s
FVC: Forced vital capacity
IQR: Interquartile range
MRI: Magnetic resonance imaging
SD: Standard deviation
TDT: Transfusion-dependent thalassemia
TLC: Total lung capacity
VA: Alveolar volume
