# Supplementary material for: Pulmonary function in patients with transfusion-dependent thalassemia and its associations with iron overload
Source: Sci Rep. 2023 Mar 4;13:3674. doi: 10.1038/s41598-023-30784-9 (PMC9985598; doi:10.1038/s41598-023-30784-9)
Supplement: Supplementary file 1 — Supplementary Tables. [file 41598_2023_30784_MOESM1_ESM.docx]

**Supplementary Table 1. Between-group comparisons of clinical characteristics of participants with available MRI measurements**

| Characteristics | Normal (n = 29) | Restrictive (n = 14) | *P* |
| --- | --- | --- | --- |
| Age (years), mean ± SD | 24.4 ± 7.3 | 24.2 ± 5.8 | 0.91 |
| Sex, n (%) | F: 15 (51.7%)  M: 14 (48.3%) | F: 5 (35.7%)  M: 9 (64.3%) | 0.35 |
| Weight (kg) | 44.8 (40.9-50.1) | 52.2 (46.6-54.0) | 0.04 |
| Height (cm) | 154.3 (148.8-159.6) | 162.1 (157.7-163.8) | 0.004 |
| BMI (kg/m^2^), mean ± SD | 19.3 ± 2.0 | 19.7 ± 2.3 | 0.61 |
| Age at diagnosis (years) | 0.8 (0.5-1.2) | 0.5 (0.2-3.0) | 0.96 |
| Age of starting blood transfusion (years) | 1.0 (0.5-2.5) | 1.2 (0.5-3.6) | 0.71 |
| Duration of blood transfusion (years), mean ± SD | 23.1 ± 7.4 | 21.8 ± 6.5 | 0.59 |
| Serum ferritin (pmol/L) | 4407.0 (2241.0-8031.5) | 4081.0 (2338.8-6054.8) | 0.59 |
| FVC (% predicted), mean ± SD | 93.7 ± 7.8 | 73.0 ± 7.9 | <0.001 |
| FEV1 (% predicted), mean ± SD | 94.2 ± 8.2 | 75.2 ± 7.5 | <0.001 |
| FEV1/FVC (%) | 88.0 (85.0-93.0) | 90.5 (89.0-92.3) | 0.16 |
| FEV1/FVC (% predicted) | 101.0 (95.5-105.0) | 103.0 (100.8-106.3) | 0.10 |
| TLC (% predicted) | 92.0 (85.0-101.0) | 69.5 (66.5-79.3) | <0.001 |
| DLCO/VA (% predicted) | 112.0 (99.5 – 128.5) | 131.0 (109.0-137.5) | 0.11 |
| MRI cardiac T2* relaxation time (ms), mean ± SD | 40.1 ± 18.2  (N= 29) | 26.5 ± 13.2  (N= 14) | 0.02 |
| MRI liver T2* relaxation time (ms), median (IQR) | 5.1 (2.2-10.9)  (N= 24) | 3.8 (2.4-12.0)  (N= 9) | 0.77 |

Values are presented as medial and interquartile range (IQR) unless otherwise specified.

P values by t-test, Mann-Whitney U and chi square test for parametric, non-parametric and categorical variables respectively

BMI: body mass index; SD: standard deviation; FVC: forced vital capacity; FEV1: forced expiratory volume in 1 second; TLC: total lung capacity; DLCO: diffusing capacity of the lung for carbon monoxide; VA: alveolar volume; MRI: magnetic resonance imaging

**Supplementary Table 2. Comparisons of clinical characteristics between participants who underwent lung function assessment 13 years ago and those who did not**

| Characteristics | With longitudinal lung function data | | *P* |
| --- | --- | --- | --- |
|  | Yes (N=23) | No (N=78) |  |
| Age (years), mean ± SD | 24.6 ± 8.7 | 26.8 ± 3.6 | 0.26 |
| Sex, n (%) | F: 10 (43.5%)  M: 13 (56.5%) | F: 39 (50%)  M: 39 (50%) | 0.64 |
| Weight (kg) | 47.0 (43.0-51.8) | 48.3 (41.3-53.1) | 0.92 |
| Height (cm) | 155.0 (147.2-163.7) | 155.6 (149.0-161.1) | 0.89 |
| BMI (kg/m^2^), mean ± SD | 20.0 ± 2.6 | 20.2 ± 2.0 | 0.72 |
| Age at diagnosis (years) | 0.8 (0.5-3.0) | 0.7 (0.4-1.6) | 0.15 |
| Age of starting blood transfusion (years) | 2.0 (0.5-3.0) | 1.0 (0.5-3.0) | 0.27 |
| Duration of blood transfusion (years), mean ± SD | 22.9 ± 8.8 | 24.8 ± 3.9 | 0.30 |
| Serum ferritin (pmol/L) | 5033.0 (2114.0-15129.0) | 3662.0 (2521.8-5984.8) | 0.12 |
| FVC (% predicted), mean ± SD | 84.2 ± 12.2 | 86.1 ± 11.9 | 0.51 |
| FEV1 (% predicted), mean ± SD | 84.1 ± 12.7 | 85.5 ± 11.5 | 0.65 |
| FEV1/FVC (%) | 88.0 (85.0-90.0) | 90.0 (85.8-93.0) | 0.12 |
| FEV1/FVC (% predicted) | 100.0 (98.0-103.0) | 102.0 (96.0-106.0) | 0.15 |
| TLC (% predicted) | 85.0 (77.0-94.0) | 85.5 (77.0-96.0) | 0.93 |
| DLCO/VA (% predicted) | 124.0 (106.0 – 142.0) | 118.0 (101.5-134.0) | 0.39 |
| MRI cardiac T2* relaxation time (ms), mean ± SD | 32.3 ± 17.8  (N= 13) | 41.1 ± 17.5  (N= 34) | 0.14 |
| MRI liver T2* relaxation time (ms), median (IQR) | 3.2 (1.4-8.4)  (N= 13) | 6.0 (2.4-14.7)  (N= 23) | 0.08 |

Values are presented as medial and interquartile range (IQR) unless otherwise specified.

P values by t-test, Mann-Whitney U and chi square test for parametric, non-parametric and categorical variables respectively

BMI: body mass index; SD: standard deviation; FVC: forced vital capacity; FEV1: forced expiratory volume in 1 second; TLC: total lung capacity; DLCO: diffusing capacity of the lung for carbon monoxide; VA: alveolar volume; MRI: magnetic resonance imaging

**Supplementary Table 3. Multivariable logistic regression analysis for the associations between variables and restrictive lung function deficit with MRI cardiac T2* as a continuous variable**

| Variables | B | SE | Exp(B) | 95% confidence interval for Exp (B) | | p |
| --- | --- | --- | --- | --- | --- | --- |
|  |  |  |  | Lower | Upper |  |
| MRI cardiac T2* | -0.06 | 0.03 | 0.94 | 0.89 | 0.99 | 0.02 |
| Male gender | 0.17 | 0.76 | 1.19 | 0.27 | 5.21 | 0.82 |
| Age | -0.02 | 0.06 | 0.98 | 0.87 | 1.09 | 0.67 |
| BMI | 0.23 | 0.19 | 1.25 | 0.86 | 1.83 | 0.25 |

**Supplementary Table 4. Multivariable logistic regression analysis for the associations between variables and restrictive lung function deficit with MRI cardiac T2* as a categorical variable**

| Variables | B | SE | Exp(B) | 95% confidence interval for Exp (B) | | p |
| --- | --- | --- | --- | --- | --- | --- |
|  |  |  |  | Lower | Upper |  |
| Abnormal MRI cardiac T2* (<=20ms) | 0.59 | 0.95 | 1.80 | 0.28 | 11.6 | 0.54 |
| Male gender | 0.56 | 0.69 | 1.74 | 0.45 | 6.69 | 0.42 |
| Age | -0.01 | 0.05 | 1.00 | 0.90 | 1.11 | 0.94 |
| BMI | 0.11 | 0.17 | 1.12 | 0.79 | 1.57 | 0.53 |

**Supplementary Table 5. Multivariable logistic regression analysis for the associations between variables and restrictive lung function deficit with MRI liver T2* as a categorical variable**

| Variables | B | SE | Exp(B) | 95% confidence interval for Exp (B) | | p |
| --- | --- | --- | --- | --- | --- | --- |
|  |  |  |  | Lower | Upper |  |
| Abnormal MRI liver T2* (<=6.3ms) | 0.51 | 0.88 | 1.67 | 0.30 | 9.36 | 0.56 |
| Male gender | 0.75 | 0.84 | 2.11 | 0.41 | 10.92 | 0.37 |
| Age | -0.06 | 0.07 | 0.94 | 0.83 | 1.08 | 0.39 |
| BMI | -0.02 | 0.24 | 0.98 | 0.61 | 1.57 | 0.93 |

**Supplementary Table 6. Multivariable logistic regression analysis for the associations between variables and restrictive lung function deficit with serum ferritin concentrations as a categorical variable**

| Variables | B | SE | Exp(B) | 95% confidence interval for Exp (B) | | p |
| --- | --- | --- | --- | --- | --- | --- |
|  |  |  |  | Lower | Upper |  |
| Ferritin <=2500 pmol/L | Reference | | | | | |
| Ferritin 2501-5000 pmol/L | -0.27 | 0.58 | 0.76 | 0.24 | 2.40 | 0.65 |
| Ferritin >5000 pmol/L | 0.59 | 0.57 | 1.81 | 0.59 | 5.51 | 0.30 |
| Sex | 0.17 | 0.45 | 1.19 | 0.50 | 2.85 | 0.70 |
| Age | 0.002 | 0.03 | 1.00 | 0.94 | 1.07 | 0.95 |
| BMI | 0.15 | 0.10 | 1.16 | 0.95 | 1.41 | 0.15 |
