# Supplementary material for: Pulmonary function in patients with transfusion-dependent thalassemia and its associations with iron overload
Source: Sci Rep. 2023 Mar 4;13:3674. doi: 10.1038/s41598-023-30784-9 (PMC9985598; doi:10.1038/s41598-023-30784-9)
Supplement: Supplementary file 2 — Supplementary Figures. [file 41598_2023_30784_MOESM2_ESM.docx]

Supplementary Figure 1. Flow diagram of the patients enrolled in the study and the subgroup of patients who had previous lung function assessment

Patients enrolled in the study (n = 101)

Patients with lung function assessments (n = 101)

Subgroup of patients who had lung function assessment performed 13 years ago (n = 23)

Patients with MRI assessments within 12 months from the lung function measurements:

- MRI cardiac T2* relaxation time (N=47)
- MRI liver T2* relaxation time (N=36)
